# Establishing the reliability and validity of the Zagazig Depression Scale in a UK student population: an online pilot study

**DOI:** 10.1186/1471-244X-10-107

**Published:** 2010-12-10

**Authors:** Ahmed K Ibrahim, Shona J Kelly, Emily C Challenor, Cris Glazebrook

**Affiliations:** 1 Community Health School, Faculty of Medicine, Assiut University, Assiut, Egypt; 2 Division of Epidemiology, Community Health Sciences School, D Floor, West Block, Queens Medical Centre, University of Nottingham, Nottingham, UK; 3 Centre for Intergenerational Health Research, University of South Australia, Division of Health Sciences, Social Epidemiology Unit, City East Campus, Adelaide, Australia; 4 Division of Psychiatry, Community Health Sciences School, A Floor, South Block, Queens Medical Centre, University of Nottingham, Nottingham, UK

## Abstract

**Background:**

It is thought that depressive disorders will be the second leading cause of disability worldwide by 2020. Recently, there is a steady increase in the number of university students diagnosed and treated as depression patients. It can be assumed that depression is a serious mental health problem for university students because it affects all age groups of the students either younger or older equally. The current study aims to establish the reliability and validity of the Zagazig Depression scale in a UK sample.

**Methods:**

The study was a cross-sectional online survey. A sample of 133 out of 275 undergraduate students from a range of UK Universities in the academic year 2008-2009, aged 20.3 ± 6.3 years old were recruited. A modified back translated version of Zagazig Depression scale was used. In order to validate the Zagazig Depression scale, participants were asked to complete the Patient Health Questionnaire. Statistical analysis includes Kappa analysis, Cronbach's alpha, Spearman's correlation analysis, and Confirmatory Factor analysis.

**Results:**

Using the recommended cut-off of Zagazig Depression scale for possible minor depression it was found that 30.3% of the students have depression and higher percentage was identified according to the Patient Health Questionnaire (37.4%). Females were more depressed. The mean ZDS score was 8.3 ± 4.2. Rates of depression increase as students get older. The reliability of The ZDS was satisfactory (Cronbach's alpha was .894). For validity, ZDS score was strongly associated with PHQ, with no significant difference (p-value > 0.05), with strong positive correlation (r = +.8, p-value < 0.01).

**Conclusion:**

The strong, significant correlation between the PHQ and ZDS, along with high internal consistency of the ZDS as a whole provides evidence that ZDS is a reliable measure of depressive symptoms and is promising for the use of the translated ZDS in a large-scale cross-culture study.

## Background

It is predicted that depressive disorders will be the second leading cause of disability worldwide by 2020, leading to significant impact on the burden of disease worldwide [1]. The NICE guidelines state that depression is a term which refers to a wide range of mental health disorders and it can manifest in many different ways, whether it is cognitive, physical, emotional, or behavioural [2]. Symptoms of depression include negative emotions such as anxiety, sleep disturbance, changes in appetite, concerns about physical symptoms, lack of self worth and feeling of despair. These can vary in severity, from minor negative emotions to suicidal thoughts, depending on each individual case [2].

Rates of depression appear to be influenced by many factors including methods of assessment [3,4], geographical location [3,5] and demographic factors such as socioeconomic status [5,6]. Although there has been much interest directed at studying depression in populations such as postpartum women, children, adolescents or the elderly, the issue of depression in college students has received relatively little attention in spite of evidence of a steady rise in the number of university students diagnosed and treated as depressed patients [7]. Recent studies have found rates of students scoring above the clinical cut-off for depression to vary from relatively low rates around 10% [8,9] to high rates of between 20% and 76% [10-14]. An international study of students aged 17-30 years from 23 countries (both developed and developing) reported a mean prevalence of 20% (19% for males and 22% for females). Highest rates were in Korea (44%), Taiwan (43.5%), Japan (35.5%), South Africa (33.5%), while values were lowest in Belgium (9.5%), Netherlands and Venezuela (10%). Rates of depression were found to be higher in students from a low income background [5]. The study also highlighted the importance of perceived control in the development of depressive symptoms; it was found that the more the control persons feel over their lives the more likely they are to have problem solving abilities and the lower their level of depression [15].

The cross-national differences in rates of depression may be explained by either true rate variation or differences in diagnostic threshold [16]. The Zagazig Depression Scale (ZDS) [17] an Arabic self-rating scale derived from The Hamilton structured interview [18] and is based on the Caroll Rating Scale (CRS) [19] has been used in a representative sample of Egyptian students [20]. It has the advantage of exploring symptoms in a number of domains including insomnia, agitation and anxiety and may be more sensitive to mild depression. An Egyptian study which used the measure found that 71% of all students scored above the recommended cut-off for mild depression [17], with a higher incidence of depressive symptoms found in students of moderate social class (51.7%), compared to those of high (17.5%) social class [21]. This pilot study aimed to establish the reliability of the ZDS [17] in a UK undergraduate student population, establish the concurrent validity of the ZDS by examining the association between ZDS scores and scores for the Patient Health Questionnaire [22] and Establish the construct validity by looking at the relationship between ZDS scores, gender, control and socio-economic status.

## Method

### Participants

An opportunistic sample of undergraduate students at UK universities was recruited. Inclusion criteria included; being a UK citizen, registered at a UK university and aged 18 years or over. It was estimated that a sample of 97 students was needed to give 80% power of calculation with 95% confidence level and error of 0.1. Assuming a response rate of 30-50%, approaching 275 students would give a sample of 97.

### Design

A cross-sectional design was used for this pilot study.

### Procedure

A total of 275 undergraduate students from a range of UK Universities in the academic year 2008-2009, were invited to join the online group on 3/11/08, which then prompted them to click on a link to the online questionnaire. Members of the group were then sent a reminder email on 13/11/07. On 17/11/07, the group was closed. Of the 275 students invited to participate in the survey, only 133 (48.36%) completed the survey. The study approved by the University of Nottingham Medical School Ethics Committee Ref. No. N/9/2008.

### Measures

#### Socio-economic measure

Four indices of socio-economic status were used;

i. **Postcode **was used to provide an area-based measure of social status via the Index of Multiple Deprivation (IMD) which takes into account seven small geographic areas (known as Lower Super Output Areas (LSOAs)) level domain indices of deprivation (Income deprivation, Employment deprivation, Health deprivation and disability, Education, skills and training deprivation, Barriers to housing and services, Living environment deprivation and Crime). A rank of 1 is assigned to the most deprived area and a rank of 32,482 is assigned to the least deprived area. In analysis the index score is divided by 1000 [23].

ii. **Mother and father's educational level: - **It has been suggested that educational measures have been more closely linked to disease outcome, compared to occupation and income measures [24].

iii. **Mother and father's occupational status: - **Participants selected their parents' most recent occupation from 8 broad occupational classifications (e.g. modern professional). Each classification was briefly described and illustrated with example jobs. Participants who could not identify their parents' occupational status were asked to describe the occupation, which was classified by the researcher using the *The National Statistics Socio-economic Classification *[25].

iv. **Family Affluence Scale (FAS): **Four questions about material-living standards developed for the WHO Health Behavior in School-aged Children Study to assess family wealth. A composite FAS score is calculated for each student with higher scores indicating greater affluence (range 0 to 9) [26].

#### Depressive symptoms measure

i. **The Zagazig Depression Scale (ZDS) **is an Arabic rating scale [17] uses the taxonomy of The Hamilton Depression Scale [18] to assess a wide range of depressive symptoms in a number of domains. The 52 items were based on the CRS [19] and assessed symptoms in 16 domains. The scale was translated into English and then back translated into Arabic to check the face validity of the translation. For the purpose of the UK study six questions from the original ZDS (used in the Egyptian study) were removed due to ambiguous meaning, poor discrimination after translation and scrutiny of the Egyptian data. We removed items which had low item-total correlation or performed poorly at the domain level. Some domains were combined. Each symptom item was scored 1 if present (and 12 items were reverse scored) to give a maximum score of 46 with higher scores indicating more depressive symptoms. A total score of < 10 was considered to indicate the absence of depression symptoms, 10-19 indicates mild, 20-29 indicates moderate, and ≥30 indicates severe depressive symptoms [17].

ii. **The PHQ-9 **consists of nine items. Respondents were asked to answer "not at all", "several days", "more than half the days" or "nearly every day" to each question, and the responses are given a mark of 0, 1, 2 or 3 respectively. The total maximum score for the PHQ-9 is 27, with <5 indicating no or minimal depression, 5-9 indicating mild depression, 10-14 moderate depression, 15-19 moderate to severe depression, and ≥20 signifying severe depression [22]. The validity, feasibility, and ability to detect changes in depressive symptoms has been supported in several studies [27-29]. Additionally, the PHQ-9 is increasingly being used in research, and has demonstrated superior criterion validity with respect to the diagnosis of depression compared with other established depression-screening questionnaires [30].

#### Sense of control measure

Six items scale assessing the sense of control that the respondent feels they have on their life were developed and validated by the MacArthur Foundation Network on Successful Mid-Life Development, and are rated "strongly agree" (1), "agree" (2), "neutral" (3), "disagree" (4) or "strongly disagree" (5), with scores ranging from a possible 6 to 30. The cronbach's alpha for the sense of control scale was .64 [31].

### Statistical analysis

The data were analyzed by using SPSS.PC (15.0). Seven participants failed to complete all the ZDS items, where 6 individuals missed a single item and one missed 2 items. This resulted in 8 missing items with no item having more than one missing value. The answers to these missing questions were then filled in using the 'Replace of missing values' option in SPSS, using the series median value. It was proposed that for purposes of univariate analysis replacing missing values can reduce bias and often is used for this purpose if data are missing at random [32]. Kappa analysis [33] was calculated to explore the degree of agreement between the ZDS and PHQ (concurrent validity). According to Fleiss; kappa over .75 is considered as excellent, .40 to .75 as fair to good, and below .40 as poor[34]. Scale reliability was then performed using Cronbach's alpha to see if individual items from both the ZDS and FAS are consistent for each scale, and to look at homogeneity. According to Bowling [35] an alpha of 0.5 or higher is considered as a sign of acceptable internal consistency. To examine construct validity the total ZDS scores were correlated with sense of control, SES measures and gender differences were tested using chi-square test. Confirmatory factor analysis was used to test how well the ZDS items represent the number of domains included.

## Results

**Figure 1:**
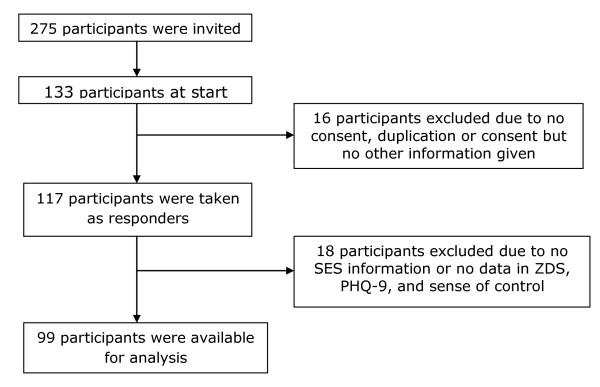
**Selection and exclusion of participants**.

Of the 275 participants approached to take part in the study, 133 (48.8%) participants completed the survey. A further 34 participants were excluded (see Figure 1) giving a usable sample of 99 (35%) to be included in the analysis. Of the 99 participants, 68.3% were aged 20 years or younger (with mean age of 20.3 years), 42.4% were male. The majority (84.4%) of participants were also rated as having high family affluence on the FAS.

### The Psychological Measures

The mean score for the ZDS was 8.3 (SD = 6.4), median was 6 and ranged from 0 to 39.

Females had higher ZDS scores (mean 9.18, SD = 6.03) than males (mean 7.17, SD = 6.86) but this difference failed to reach significance (p > 0.05). Females (38.6%) were, however, significantly more likely to score above the cutoff for depression compared to males (19%) (χ^2 ^= 4.6, df = 1, p = 0.03) (Table 1).

**Table 1:** Zagazig and PHQ severity by gender

		Male (N = 42)	Female (N = 57)	Total (N = 99)
**Zagazig Severity**	None (< 10)	34 (81.0%)	35 (61.4%)	69 (69.7%)
	Mild (10-19)	6 (14.2%)	18 (31.6%)	25 (25.3%)
	Moderate (20-29)	1 (2.4%)	4 (7.0%)	4 (4%)
	Severe (≥30)	1 (2.4%)	0 (0.0%)	1 (1%)

**PHQ Severity**	None (< 5)	33 (78.6%)	29 (50.9%)	62 (62.6%)
	Mild (5-9)	5(11.9%)	20 (35.1%)	25 (25.3%)
	Moderate (10-14)	3 (7.1%)	7 (12.3%)	10 (10.1%)
	Moderate to severe (15-19)	0(0.0%)	1 (1.8%)	1 (1%)
	Severe (≥20)	1 (2.4%)	0 (0.0%)	1 (1%)

**Figure 2:**
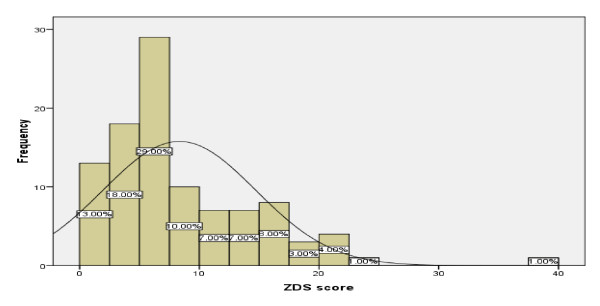
**Histogram for Zagazig score**.

The distribution of ZDS scores is shown in Figure 2. The data are positively skewed (skewness = 1.6, SE = .24) and flatter than normal (kurtosis = 4.3, SE = .5), showing that the majority of participants had no symptoms or only a mild depressive symptoms.

### Reliability of ZDS

When scale reliability was performed on the whole 46-item ZDS with the 16 domains, it was found Cronbach's alpha = .894, which shows there is very good consistency between the individual items in the ZDS. There are no individual questions in the ZDS which, if deleted, would improve Cronbach's alpha. There are also no individual questions which, if deleted, would worsen Cronbach's alpha substantially. This shows that there is good overall consistency with each component of the ZDS.

Table 2 demonstrates the Cronbach's alpha for each domain in the ZDS. For the purpose of analysis some domains addressing the same concept were added up; (insomnia early, middle and late added up together to be insomnia), (anxiety psychological and somatic added up to be anxiety) and (GIT symptoms, libido, general and Hypochondrisis added up in General). According to Bowling [35] an alpha of 0.5 or higher is considered as a sign of acceptable internal consistency. For depressed mood, Cronbach's alpha = .596, which was acceptable, and did not improve if you remove any of the individual questions from the domain. For feelings of guilt, Cronbach's alpha = .532, which was acceptable. For suicide, Cronbach's alpha = .817, which was very good. However Cronbach's alpha would be 1.00 if Q37 (life is worth living) was deleted. This was because there were exact answers for Q20 and Q30. All answers for both Q20 and Q30 were 'no', apart from 1 person who answered 'yes' to both. This could hint that there was a problem with multicollinearity or singularity for this domain. For the insomnia domain, Cronbach's alpha = .758, which was good. This would not improve if any of the questions were removed.

**Table 2:** Cronbach's alpha for depression domains of ZDS

Domain	Cronbach's Alpha*	N of Items
**Depressed mood**	.596	4

**Feelings of guilt**	.532	4

**Suicide**	.817	2

**Insomnia (3)**	.758	5

**Work and activity**	.618	4

**Retardation**	.531	4

**Anxiety (2)**	.709	9

**Agitation**	.370	4

**General (4)**	.562	8

**Weight loss**	.471	2

*Based on Standardized Items

For work and activity, Cronbach's alpha = .618, which was average, and would not improve if any of the questions were removed. For retardation, Cronbach's alpha = .531, which was acceptable, and would not improve if any of the questions were removed. For agitation, Cronbach's alpha = .370, which was poor, and would not improve if any of the questions were removed. For anxiety Cronbach's alpha was .709, which is good. For general symptoms it was .562, which was acceptable. For loss of weight Cronbach's alpha was .471, which is poor. However, alpha can't be accurately calculated form domains with only 2 items.

There was a strong association between ZDS scores and PHQ scores (Spearman's Rho = 0.795, p < .0001). Using the recommended cut-offs for the ZDS and the PHQ to classify participants as above minimum threshold for depression there was agreement on whether the participant was depressed (28.7%) or not depressed (60.4%) for 91.1% of cases. In only 2 cases (2%) were participants classified as depressed by the ZDS and not by the PHQ (Table 3). The resulting Kappa score was 0.76 (p < 0.001) which approaches very good agreement [36].

**Table 3:** ZDS vs. PHQ cross tabulation

		PHQ		Total
		Not depressed	Depressed	
**ZDS**	**Not depressed**	60 (60.4%)	9 (8.9%)	69 (69.3%)
	
	**Depressed**	2 (2%)	28 (28.7%)	30 (30.7%)

**Total**		62 (62.4%)	37 (37.6%)	99 (100%)

To explore agreement on level of depression a weighted kappa was calculated. There was high agreement between both scales regarding severity (83.1%). The weighted Kappa was 0.678 (p < 0.001) indicating good agreement [36]. There was also a strong positive association, with a correlation between levels of depression as assessed by the 2 scales (r = .81, p < .001) (Table 4).

**Table 4:** Grades of severity of depression in ZDS vs. PHQ cross tabulation

		PHQ				Total
		No	Mild	Moderate	Severe	
**ZDS**	**No**	60 (60.4%)	8 (7.9%)	1 (1%)	0 (0%)	69 (69.3%)
	
	**Mild**	2 (2%)	17 (17.8%)	5 (5%)	0 (0%)	24 (24.8%)
	
	**Moderate**	0 (0%)	0 (0%)	4 (3.9%)	1 (1%)	5 (4.9%)
	
	**Severe**	0 (0%)	0 (0%)	0 (0%)	1 (1%)	1 (1%)

**Total**		62 (62.4%)	25 (25.7%)	10 (9.9%)	2 (2%)	99 (100%)

In order to explore the construct validity of the ZDS, scores were correlated with measures of socio-economic status and control measure (Table 5). Parental level of education was mildly correlated with ZDS scores (r = -.206, p < 0.05). Moreover, Sense of Control scores were moderately correlated with ZDS score (r = -0.573, p < .01). These findings indicated that as the level of education of parents or the sense of control decreases, the level of depressive symptoms increases. Other measures of SES (i.e. Index scores, FAS and parental occupation) were not correlated with ZDS scores (r > .2, p > 0.01).

**Table 5:** Spearman's correlation between Zagazig, SES and Control scores

	Number (N = 99)				
	**Zagazig Score**	**Index score**	**FAS**	**Parents' education**	**Parents' occupation**

**Zagazig Score**	1.000				

**Index score**	.102** (> 0.01)	1.000			

**FAS**	-.142**(> 0.01)	-.092**(> 0.01)	1.000		

**Parents' education**	-.206*(< 0.05)	-.022**(> 0.01)	.170**(> 0.01)	1.000	

**Parents' occupation**	-.119**(> 0.01)	-.188**(> 0.01)	.142**(> 0.01)	.070**(> 0.01)	1.000

**Control Score**	-.573**(< 0.01)	-.151**(> 0.01)	-.153*(> 0.05)	.152**(> 0.01)	.010**(> 0.01)

* Correlation is significant at the 0.05 level (2-tailed).

** Correlation is significant at the 0.01 level (2-tailed).

## Discussion

In this sample 30.3% of participants were classified as depressed as measured by the ZDS, and 37.4% as measured by the PHQ-9. These levels are relatively high compared to the general population, where it is thought that about 6% to 20% of people suffer from depression [37,38]. This high prevalence is consistent with previous analysis of depression in university students [20,21] but other studies, using a number of different depression scales, have found much lower levels of depressive symptoms [22,23].

Regarding the Zagazig Depression Scale as an accurate measure of depressive symptoms, it was found that the ZDS score was very similarly distributed to the PHQ score. Both were positively skewed (indicating that few students suffered from moderate or severe depressive symptoms), and there was no significant difference between the PHQ and ZDS scores. The correlation between the two was strong and positive, suggesting that the ZDS is a reliable measure of depressive symptoms in the UK sample of students studied.

According to the data collected in this study, there was a significant (p < 0.05) difference between gender and the severity of both the Zagazig and PHQ depression scores (Table 1). These findings are not surprising, since it is reported by NICE that each year 1 woman in 15, compared to one man in 30 is affected by depression each year. This Office for National Statistics supports the idea that more women suffer from affective disorders than men [2]. The increased prevalence of depression in women compared to men has been reported in studies which looked at depression in the general population [24,25]. In cross-national depression research using the M-BDI in university students, Mikolajczyk et al. (2008) [39] also found female students in Germany, Denmark, Poland and Bulgaria suffered more depressive symptoms than men in all countries, while the findings of Dahlin et al. (2005) [40] showed that the female medical students in their cross-sectional study were almost 2.5 times more likely to suffer from depressive symptoms (measured by the MDI) than the male students. This is consistent with other cross-national and UK-based surveys [28,29].

A very small proportion of the sample suffered from severe depressive symptoms, with 5% (according to ZDS) and/or 12% (according to PHQ-9) of the sample suffering from moderate or severe depression. Kessler et al. also found that students more likely to suffer from minor but not major depression, using the CIDI (supplemented by DSM-III-R criteria) [41]. Wong et al. (2006) found that mild and moderate depression rates 14.2% and 12.9% respectively in a sample of first-year tertiary education students in Hong Kong, compared to 5.0% experiencing severe and 3.0% extremely severe depression using the Depression Anxiety Stress Scale, [42]. More analysis of depressive severity in UK students is necessary to determine whether my data is consistent with other studies in the UK population. These findings may demonstrate that the symptoms may be anxiety and stress-related, rather than actual symptoms of depression. Although the PHQ-9 is a frequently-used, accurate measure of depression, it doesn't differentiate between symptoms of anxiety which is characterized by chronic worry about all sorts of life problems and circumstances and symptoms of depression which cover a very wide range of problems, from short periods of low mood to a lifetime of mind-numbing inability to function. It is likely that people with clinical depression will also have anxiety disorder [43]. An advantage of the ZDS is that it taps a broader range of symptoms and may thus be more sensitive to mild depression.

Student-related stress is a common idea, where workload, moving away from home and money problems may add extra stress to an individual, without actual depression being present. Mikolajcyzk et al. [39] reported that some of the main somatic symptoms of depression, such as disrupted sleep and eating patterns, may not be indicative of depression, but illustrate the disturbance university-related factors impose on the student. Other studies have found similar levels of increased stress and anxiety in university students [20,44]. A classic example of this is a disrupted sleep cycle in the lead-up to a major exam. For this reason, questions such as 'I can concentrate easily without problems' and 'I wake up in the early hours of the morning and cannot get back to sleep again' may reflect the stress and anxiety which students undergo, and not actual depressive symptoms.

Some of the questions in the ZDS seemed to ask the same question, such as 'life is worth living' (question 37) and 'life is not worth living' (question 30). This may lead to confusion and the questions being answered inaccurately, although after cross tabulation, some questions were answered consistently. However, a number of questions were also answered inconsistently, indicating that they should perhaps be removed from the larger-scale study. For example, 'life is not worth living' and 'life is worth living' were only consistent by 0.398, suggesting these questions need to be reviewed.

The sense of control score was slightly negatively skewed, indicating that more individuals in the sample had a high sense of control. The moderate, negative correlation between the sense of control score and both measures of ZDS and PHQ score indicates that increasing sense of control is associated with decreasing depressive symptoms. This is consistent with previous work, which found decreasing levels of sense of control are significantly associated with increasing rates of depression [31,5] and is consistent with increasing control with increasing SES [31,45] so may reflect the relatively high SES of this study population.

The scale reliability of the FAS demonstrated that the individual measures of the FAS weren't consistent for our sample. As the FAS has been previously validated [26] and used in a large number of other studies, the likely reason for poor Cronbach's alpha here is the poor heterogeneity of the study sample used. Looking at the individual components of SES, you can see that a huge number of individuals have their own bedroom (98.0%), which could explain the weak scale reliability. There are also a large number of people who reported having more than two computers (73.7%). The computer question could be a problem, especially in recent times with new technology, as many people buy new computers but keep their old ones. The report of how many computers they have may not reflect how many computers are used in the household, therefore the main study may benefit from using the rephrased question of 'How many computers are in use in your household?'

There is also the issue of whether the FAS which is a useful marker for SES in university students as it was developed for children. More detailed examination of the FAS and HBSC 2005/06 survey has indicated that older children are more likely to have their own bedroom (independent of family wealth), have more computers, and have more cars in the family [46]. The use of FAS as a marker for SES in the current study for older participants may therefore not be reliable but will provide a supporting evidence for the SES of students.

The analysis of the individual measures of SES used in this work were not highly correlated, with only FAS score and father's education, father's and mother's education, and father's and mother's occupation being significantly correlated. The reasons behind this could be due to the fact that some people did not select the correct occupational class. While correlation between the markers for SES is important for sociologists we do already know that the relationship between the different components of SES and depression is much more complex. The magnitude of the relationship between socioeconomic status and depression depends on which variable is included in the model, and previous work has shown that multiple elements of social class are needed to predict its relationship with depression [6,47]. However, Hudson (2005) found that an inverse depression-SES gradient was illustrated regardless of which measure of SES was used [48] with only the magnitude of the association changing.

Scale reliability found the ZDS to have a Cronbach's alpha of 0.894, which is nearly excellent [49]. None of the individual questions would worsen or improve this value if they were deleted, which shows very good overall consistency with each component of the ZDS. The internal consistency in the pilot study is similar to that found in the Egyptian study, where Cronbach's alpha was excellent (0.904) [21].

Factor analysis demonstrated fair loading of variables for the depressed mood, feeling of guilt and suicide factors, however the loading was not satisfactory for the rest of factors in the modified ZDS, while the loading was better in the Egyptian study (for all domains except retardation, somatic anxiety and libido domain), this again highlights how students in different countries will display different depressive characteristics.

Although the ZDS was developed from the Hamilton Depression Scale (via the Caroll scale), a very widely known and used depression measure at the time of development of the original ZDS [50], it has been translated from Arabic, so some problems involving cultural differences between depression in the UK and Egyptian student population may exist. For example, 'I think I am a hopeless case' and 'I think I have serious diseases', which are questions some people missed out in the original data collection, may not quite convey the same meaning as if they were written in Arabic or they may be detached from their literal meaning.

The design of the main questionnaire may need to be re-considered to allow people to only answer the questionnaire once in the future study. The deletion of some of the participants, due to non-response to a number of crucial questions also needs to be examined. The larger-scale survey may benefit from only allowing people to proceed with the survey if they have filled in every question. This may, however, discourage some people from answering the survey at all.

### Limitations of the study

The response rate was 49%, with a usable response rate of 36%, which could be considered low. This is in line with rates found in previous online surveys which have ranged between 30 to 50% [51]. A low response rate is problematic as non-respondents may differ from respondents in other respects than just their willingness to participate in a survey [52,53]. The students in this pilot study were predominantly drawn from higher social classes with 84% classified as high on the Family Affluence Scale and 64% with fathers with degree level education. As higher social class is associated with lower levels of depression it may be that this survey underestimates the level of depression in the student population and the ZDS may only be valid and reliable in this population. There is also a possibility that males are underrepresented in this sample (42.4%). However UK university statistics show that there is a steady increase in the proportion of female students so that they now outnumber males [54].

The evidence for construct validity is mixed. There is a moderate relationship between control and ZDS (r = 0.57) and women were more likely to be classified as depressed as expected. However, the predicted relationship between higher ZDS scores and lower social class, although statistically significant, is weak. This probably reflects the homogeneity of social class in this sample. The survey was anonymous to encourage honest reporting of symptoms but, consequently, it was not possible to assess the test-retest reliability of the ZDS in this sample. This omission will be addressed in the main study by asking a subset of responders to complete the ZDS at a second time point.

## Conclusion

The current study has provided a good basis on which the main study, also an online survey, can be built. It has highlighted individual problems which might arise in using the ZDS on the UK student population, and perhaps questioned the use of the FAS as a measure of SES. It confirms that multiple measures of SES should be used to ensure a measure of socio-economic status. The strong, significant correlation between the PHQ and ZDS, along with high internal consistency of the ZDS as a whole is a promising for the use of the translated ZDS in the UK. The main study will build on the current study, where a larger sample drawn from universities serving students from a wider range of social backgrounds will be used, and a link between the socio-demographic variables and depressive outcomes will hopefully be established. The universities can then use the information and findings from the main study to help individuals which may be flagged up as experiencing severe depression, if those individuals seek it.

## List of abbreviations

**ZDS**: Zagazig Depression Scale; **NICE**: National Institute of Health and Mental Excellence; **CRS**: Carroll rating scale; **PHQ-9**: Patient Health Questionnaire, 9-question version; **IMD**: Index of Multiple Deprivation; **LOSA**: Lower Super Output Area; **FAS**: Family Affluence Scale; **SPSS**: Statistical Package of Social Sciences; **CIDI**: Composite International Diagnostic Interview; **DSM**: Diagnostic and Statistical Manual of Mental Disorders; **BDI**: Beck Depression Inventory; **SES**: Socio Economic Status; **HBSC**: Health Behaviour in School-Aged Children.

## Competing interests

The authors declare that they have no competing interests.

## Authors' contributions

All authors contributed equally to this work. They have read and approved the final draft.

## Pre-publication history

The pre-publication history for this paper can be accessed here:

http://www.biomedcentral.com/1471-244X/10/107/prepub
